# A Case of an Oral Elastofibromatous Lesion: A Clinicopathological Analysis With a Literature Review

**DOI:** 10.1155/crid/5556176

**Published:** 2025-01-11

**Authors:** Sawako Ono, Masanori Masui, Kyoichi Obata, Tomoya Nakamura, Yoshihiko Furuki, Satoko Nakamura, Hidetaka Yamamoto

**Affiliations:** ^1^Department of Pathology and Oncology, Graduate School of Medicine, Dentistry and Pharmaceutical Sciences, Okayama University, Okayama, Japan; ^2^Department of Oral and Maxillofacial Surgery, Kagawa Prefectural Central Hospital, Takamatsu, Kagawa, Japan; ^3^Department of Oral and Maxillofacial Surgery, Graduate School of Medicine, Dentistry and Pharmaceutical Sciences, Okayama University, Okayama, Japan; ^4^Department of Pathology, Kagawa Prefectural Central Hospital, Takamatsu, Kagawa, Japan

**Keywords:** elastofibroma, oral elastofibromatous lesion, oral mucosa

## Abstract

Elastofibromatous changes of the oral mucosa, such as an elastofibroma (EF) or an elastofibromatous lesion (EFL), are not well recognized, and the second such case in Japan is reported. A 72-year-old man wearing a complete maxillary denture presented with a small nodule on the hard palate. Histopathological examination showed abundant fibrous tissue with numerous elastic fibers on Elastica van Gieson (EvG) staining. The diagnosis of an oral EFL was made. In the review of oral EF and EFL, no cases with recurrence were identified, but such lesions may resemble neoplastic lesions macroscopically. Accurate diagnosis using EvG stain is needed to recognize oral EFs and EFLs.

## 1. Introduction

An elastofibroma (EF) is a benign, ill-defined proliferation of fibroelastic tissue with abnormal elastic fibers, and an elastofibromatous lesion (EFL) is reported to be a precursor of EF [1, 2]. EFs and EFLs have been reported in regions other than the lower scapula and thoracic wall, including the neck, mediastinum, gastrointestinal tract, and multiple sites in subcutaneous tissue [3–10]. However, EFs and EFLs of the oral mucosa are not well recognized. A rare case of an oral EFL on the hard palate is reported along with a literature review to further characterize the clinicopathological features of oral EFs and EFLs.

## 2. Case Presentation

**Figure 1:**
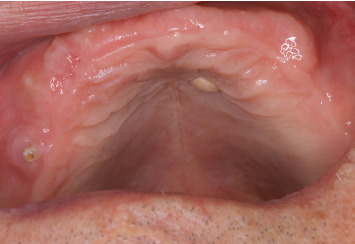
Clinical findings. A solitary raised lesion with a smooth surface is visible on the hard palate.

**Figure 2:**
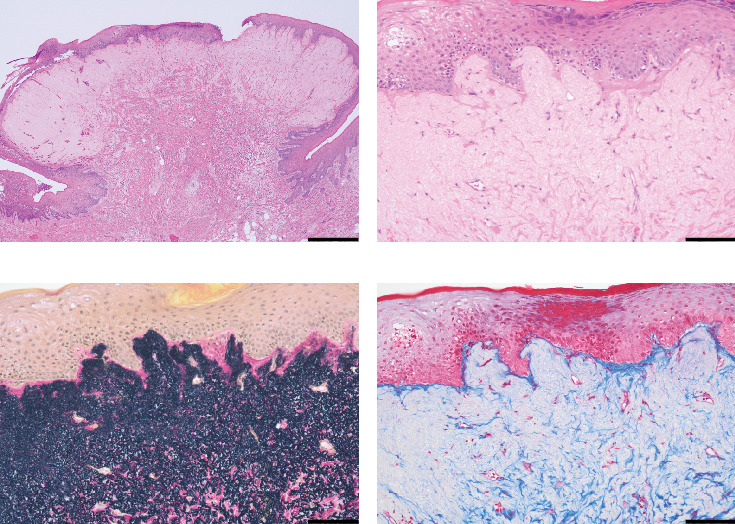
(a–d) Hematoxylin–eosin (HE) stain. Bars: (a) 500 and (b–d) 100 *μ*m. (a) Histological examination shows a raised nodular growth covered by stratified squamous epithelium. (b) Proliferation of pale eosinophilic fibers is observed. (c) Elastica van Gieson stain shows dense and prominent elastic fibers, (d) but Masson stain shows sparse collagen fibers.

A 72-year-old man with no history of cancer and no relevant family history was found to have a mass on the hard palate during a medical examination. On his visit to our hospital, an approximately 5 mm submucosal mass was palpated on the left side of the hard palate, and the overlying epithelium of the mass appeared healthy on intraoral examination (Figure 1). A complete denture was used in the maxilla. Computed tomography (CT) showed no maxillary destruction. Clinically, the patient was diagnosed as having fibrous hyperplasia, and the lesion of the hard palate was resected. The surgically removed lesion measured 4 × 2 mm^2^. The gross appearance of the cut surface of the excised lesion showed a white elevated nodule. Histopathological examination showed the proliferation of pale fibrous tissue within the submucosa and dense eosinophilic fibrous tissue at the base (Figure 2). Elastica van Gieson (EvG) staining showed a high density of elastic fibers covered by mucosal epithelium, with few collagen fibers in the submucosa. Alcian Blue staining showed no mucin retention. There was no evidence of malignancy. These findings suggested the diagnosis of an oral EFL. Postoperatively, no additional therapy was given to the patient. Three months after the surgery, no recurrence was observed.

## 3. Discussion

An EF is defined as an ill-defined proliferation of fibroelastic tissue, and an EFL has been reported to be an incipient elastofibromatous degenerative process that has not yet reached the appearance of a fully developed EF [1, 2]. An oral EF was first described by Potter, Summerlin, and Rodgers in 2004, after which several reports of oral EFLs followed [11]. To the best of our knowledge, there have been reports of elastic fiber disorders of the oral mucosa from the United States and Europe, but this is the second report from Asia in the English-language literature [11–18]. A total of 15 cases were analyzed (Table 1), with eight classified as EFs and seven as EFLs. The mean age for the EF cases was 58.2 years, with a predominance of males. The palate was the most common site, and the average size was 4.6 mm. In contrast, the mean age for the EFL cases was 73.1 years, with slightly more females. The most common sites were the alveolar mucosa and palate, and the mean size was 7.4 mm. All 15 cases involved only a single lesion. No recurrence was reported in patients for between 3 and 120 months. Rodrigues Rodrigues et al. in Brazil reported an overview of 77 cases of oral EFs and EFLs [19]. They reported that the mean age was 39 years for EFs and 30 years for EFLs. The buccal mucosa was the most common area affected in both types, and the mean size was 9.9 mm for EFs and 7.8 mm for EFLs. Compared with the present study, they reported an extremely large number of cases, younger age of occurrence, and slightly larger size. Regional factors may be associated with the occurrence of oral EFLs and EFs.

The macroscopic findings of oral EFLs and EFs varied, including a submucosal mass, nodule, plaque, papule, slightly elevated lesion, or flat area. The color was white to yellow. The clinical diagnoses also varied, and most were non-neoplastic lesions, including fibromas, hyperkeratosis, aphthae, submucosal abscesses, lymphoid aggregates, lichen sclerosus et atrophicus, and minor salivary gland cysts [11–18]. However, neoplastic lesions such as soft tissue neoplasms including lipoma, dysplasia, and cancer were also suspected in some cases, one of which showed acute onset of a nodule [11–13, 18]. Therefore, it is difficult to suspect oral EFs and EFLs clinically, and preoperative biopsy is important to prevent overtreatment.

Histologically, the elastic fibers of EFs in soft tissue are thick or coarse, deeply eosinophilic, and fragmented into linearly arranged globular or serrated disc-like structures, simulating beads on a string [1]. In contrast, in oral EFLs, some cases showed spherical, fragmented, or globular elastic fibers, whereas in others, only aggregation and proliferation were observed [13, 14, 17, 18]. In the present case, only aggregation and proliferation of elastic fibers were observed, with no spherical or fragmented elastic fibers. Therefore, the present case was considered a degenerative process preceding EF and diagnosed as an oral EFL.

Clinically, fibrous hyperplasia was suspected in the present case. On histological examination alone, it is difficult to differentiate fibrous hyperplasia and oral EFs or EFLs. Rodrigues Rodrigues et al. reported 77 cases of oral EFs and EFLs reclassified from 95 cases primarily diagnosed as oral fibromas, and oral EFs and EFLs may be underestimated [19]. To identify oral EFs and EFLs, the pathologist should perform a histological evaluation using an EvG stain.

Oral EFs or EFLs occur in the superficial lamina propria and submucosa, and the most common suggested etiology is trauma. The oral mucosa has a layer of fibrocollagenous and elastic tissue in the lamina propria and submucosa, and their hyperproliferation and degeneration would be associated with oral EFs or EFLs. Elastin is reported to be the most abundant in the soft palate but the lowest in the hard palate [20]. However, since it occurs in areas other than the soft palate, it may be related not only to the amounts of elastic fibers in the mucosa but also to the degree of irritation applied to the mucosa.

EFs arise most often in the deep soft tissue between the lower scapula and the thoracic wall [1]. The cause is considered to be friction of the lower scapula against the thoracic wall due to repetitive minor trauma and manual labor [6]. However, EFs often show chromosomal instability, including gains of 6p25-q25 and Xq12-q22 and losses of 1p, 13q, 19p, and 22q [21, 22]. In addition, deletion of CASR (3q21), GSTP1 (11q13), and BRCA2 (13q12) and gains of APC (5q21) and PAH (12q23) were observed [22]. Whether the presence of this chromosomal instability, not usually observed in non-neoplastic tissues, suggests a possible neoplastic origin for EFs remains to be determined. EFs and EFLs in the oral mucosa have not been analyzed genetically, and future studies including genetic analyses are needed to clarify the possibility of a neoplastic origin.

## 4. Conclusion

Surgeons and pathologists need to understand that EFs and EFLs occur in the oral mucosa, and preoperative biopsy and EvG staining should be used for correct diagnosis and treatment.

## Figures and Tables

**Table 1:** Summary of previous cases of oral elastofibromas and elastofibromatous lesions.

**Author**	**Country**	**Diagnosis**	**Age (years)**	**Sex**	**Location**	**Macroscopic appearance**	**Symptoms**	**Size (mm)**	**Treatment**	**Outcome**
Potter, Summerlin, and Rodgers [11]	United States	EF	56	F	Floor of mouth	Well-circumscribed, soft-tissue, submucosal mass	No	4	S	NR
Manchandu, Foote, and Alawi [12]	United States	EF	71	M	Floor of mouth	Nodular mass	No	6	S	NR
Tosios et al. [13]	Greece	EFL	76	F	Floor of mouth	Small, flat, white area	NM	NM	NM	NM
	Greece	EFL	98	F	Alveolar mucosa	An area of leukoplakia	NM	NM	NM	NM
Nonaka et al. [14]	Brazil	EFL	55	M	Soft palate	Whitish, smooth-surfaced plaque	No	10	S	NR
Darling et al. [15]	Canada	EF	33	M	Plate	Nonulcerated lesion	No	2	S	NR
	Canada	EF	43	M	Plate	Yellow growth	No	3	S	NR
	Canada	EF	50	M	Floor of mouth	Leukoplakia	NM	7	S	NR
	Canada	EF	76	F	Lower lip	NM	NM	7	S	NR
	Canada	EF	75	M	Tongue	White nodule	NM	5	S	NR
Daley and Darling [16]	Canada	EF	62	M	Hard palate	Exophytic, smooth-surfaced, pink nodule	No	3	S	NM
Silva et al. [17]	Brazil	EFL	63	F	Buccal mucosa	Localized, slightly elevated lesion	No	10	S	NM
	Brazil	EFL	76	M	Inferior vestibular fornix	A solitary white lesion	No	10	S	NM
Ogawa et al. [18]	Japan	EFL	72	F	Alveolar mucosa	White-colored, smooth-surfaced, firm papule	No	3	S	NR
Present case	Japan	EFL	72	M	Hard palate	Submucosal mass	No	4	S	NR

Abbreviations: EF: elastofibroma, EFL: elastofibromatous lesion, NM: not mentioned, NR: no recurrence, S: surgical excision.

## Data Availability

Data supporting this case report are available from the corresponding author or first author on reasonable request.
